# Static and Dynamic Magnetic Resonance Imaging in Female Pelvic Floor Dysfunction: Correlation With Pelvic Organ Prolapse Quantification

**DOI:** 10.7759/cureus.44915

**Published:** 2023-09-08

**Authors:** Pallavi Jha, Radha Sarawagi, Rajesh Malik, Aman Kumar, K Pushpalatha

**Affiliations:** 1 Radiology, All India Institute of Medical Sciences, Bhopal, IND; 2 Obstetrics and Gynecology, All India Institute of Medical Sciences, Bhopal, IND

**Keywords:** dynamic mri, pop-q staging, mri, pelvic floor, pelvic organ prolapse

## Abstract

Background: Pelvic organ prolapse (POP) is clinically assessed and staged commonly by the pelvic organ prolapse quantification (POP-Q) system. Dynamic magnetic resonance imaging (MRI) of the pelvic floor is an emerging modality for anatomical and functional assessment of the pelvic floor and staging of POP. The purpose of this study was to correlate the dynamic MRI findings with POP-Q examination for the staging of POP in each pelvic compartment by comparing various anatomic points.

Methods: A prospective observational study of the comparative cross-sectional design was conducted among patients who underwent MRI of the pelvic floor and POP-Q at our institute. A total of 50 patients were included. Anatomical landmarks in the three compartments were analyzed in relation to standard reference lines on dynamic MRI and compared with POP-Q measurements.

Results: Most of our patients had multicompartment disease (70%). When compared to POP-Q, MRI has a strong correlation for quantification of anterior (0.723) and middle (0.525) compartments and a weak correlation (0.232) for posterior compartment prolapse.

Conclusion: POP-Q examination is based on the various points within the vaginal canal, and all the points do not represent a true anatomic landmark. MRI, on the other hand, is based on a true anatomical plane and gives detailed information about various structures in all three compartments. Thus, MRI also helps bridge the gap between various referring specialties in treating pelvic floor disorders.

## Introduction

Pelvic organ prolapse (POP) occurs due to the weakening of the various ligaments and pelvic floor support and results in the displacement of the pelvic organs from their normal anatomical positions. Pelvic floor dysfunction affects a considerable number of women worldwide. Approximately 20% of women undergo surgery for POP and/or urinary incontinence during their lifetime. The incidence increases with age and in post-menopausal women. Literature has shown an estimated prevalence of around 41-50% in elderly women, adding to their morbidity and deteriorating quality of life [1-3].

Several modifiable and unmodifiable risk factors are identified with primary POP like higher age, menopausal status, obesity, vaginal delivery, and multiparity connective tissue disorders [2,4]. Factors like poorly supervised labor, prolonged duration of labor, and heavy manual work in the postnatal period have also been shown to be associated with POP, more commonly in middle and low-income countries [5,6]. Patients present with a myriad of symptoms, such as lower back pain, heaviness, feeling of something coming out of the vagina, straining, urinary frequency, urgency and hesitancy, sexual dysfunction, and constipation [6].

POP is quantified using the pelvic organ prolapse quantification (POP-Q) system by gynecologists. It was introduced in 1996 to quantify prolapse, make management decisions, and conduct clinical research [7]. Although it is a standard technique, it is highly physician-dependent [8]. In experienced hands, it is fast and can be done in the same clinical setting. POP-Q system uses vaginal topography and nine different measurements to stage prolapse [7]. Various studies have reported inter and intraobserver variability in this technique due to its complex nature and limited usage. Despite these limitations, POP-Q is widely adopted and is considered a good standard among gynecologists [9-12].

Management of pelvic floor dysfunction depends on factors such as patient age, the severity of symptoms, sexual function, and other comorbidities. Initial management of pelvic floor dysfunction is conservative, particularly in patients with mild symptoms. Accurate diagnosis of pelvic floor abnormalities is essential in planning operative procedures to minimize the risk of recurrence and reoperation. Studies have found that the recurrence of symptoms can range from 10% to 30% and can be partly due to failure to identify all the components of the multicompartment involvement [6]. While a physical examination is a primary tool for POP evaluation, it has limitations. Determining the structure prolapsing behind the vaginal mucosa can become challenging and sometimes subjective. The reference plane, i.e., the hymen, is a relatively mobile structure that can lead to inconsistencies. The pelvic organs are not staged separately; rather, a composite staging is offered based on the maximum prolapsing structure. Various imaging procedures are used to evaluate pelvic organ dysfunction, like voiding cystourethrography, fluoroscopic cystocolpodefecography, and, recently, translabial sonography. These are practical and cost-effective modalities. However, these studies have their limitations [6].

Static and dynamic magnetic resonance imaging (MRI) of the pelvic floor provides superior soft tissue resolution and multiplanar capabilities. It thus allows better anatomical and functional evaluation of the pelvic floor. It is a multiphase study with images acquired at rest, squeeze, strain, and defecation phases. The static component of the study allows visualization of the musculoligamentous component of the pelvic floor, and the dynamic component enables the assessment of prolapse using consistent and easily identifiable anatomical landmarks as the plane of reference. The images are easier to interpret, and prolapse is easily quantifiable [13-15]. Literature has shown a high recurrence rate (32%) of POP after surgery, with a reoperation rate of 11-20% [16,17]. Accurate assessment of all pelvic floor compartments is essential in planning a surgical repair to minimize the risk of repeated surgery [18].

Despite the availability of MRI of the pelvic floor for over two decades, there has been a dearth in its clinical utilization. In this study, we evaluate the static and dynamic MRI appearance of pelvic floor dysfunction and examine the correlation between MRI and POP-Q for various pelvic floor compartments.

## Materials and methods

This observational study of the comparative cross-sectional design was conducted in a tertiary care hospital after approval from the institute's ethics committee (Institutional Human Ethics Committee (IHEC), AIIMS Bhopal; approval number: IHECPGRMD033). All adult women who presented with complaints suggestive of pelvic floor dysfunction and underwent an MRI study of the pelvic floor were considered for inclusion. Written informed consent was obtained from all the participants. Patients who did not give consent, who had a previous history of pelvic surgery, procidentia on clinical examination, and a study with poor-quality images were excluded.

**Figure 1:**
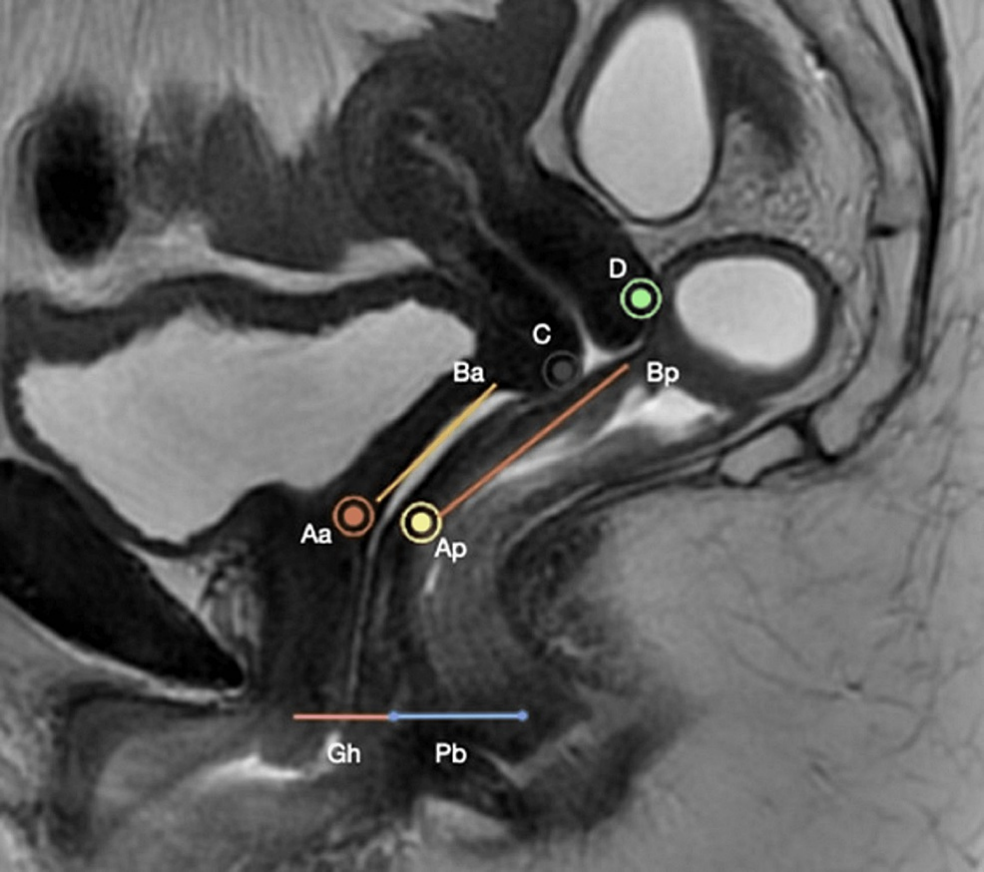
The vaginal topography used in the POP-Q system as depicted on the sagittal T2-weighted image. Note that the bladder is partially filled and the rectum is empty during the POP-Q system. The points are denoted as (+) if they are below the hymen and (-) if they are above the hymen. No sign is assigned to genital hiatus (Gh) and perineal body (Pb), as they are measured independently of the hymen. POP-Q: pelvic organ prolapse quantification.

Detailed clinical history was taken in all patients. An expert gynecologist performed a clinical examination pertinent to the pelvic floor weakness in an empty bladder using the POP-Q method [7]. After taking informed consent, the patient was examined in a lithotomy position with a partially filled bladder, and the presence or absence of cystocele was documented. The patient was then asked to empty her bladder, and an examination according to the POP-Q classification was performed. The positions of the six points in the vagina were measured using a centimeter ruler with respect to the hymen during maximal straining or cough. Points lying above the hymen were denoted with a negative sign, points at the hymen were denoted as 0, and those below the hymen were recorded as positive integers. In the end, three descriptive measurements were recorded independent of the hymen: genital hiatus (GH) point, perineal body (PB) point, and total vaginal length (TVL) at rest point. TVL is recorded at rest, with the prolapse reduced (Figure 1).

The above measurements were recorded in a tic-tac-toe grid. The anterior vaginal wall points were described in the top row, and the posterior vaginal wall and posterior fornix were on the bottom row. The measurements of GH, TVL, and PB were recorded in the middle row. Using the POP-Q system, the prolapse of each compartment is staged based on the relationship with the hymen. Points Aa and Ba were used to determine the stage of the anterior compartment. Points C and D were used to stage the middle compartment, and Ap and Bp were used for the posterior compartment. The overall stage was also assigned, taking into consideration the leading edge.

These patients underwent MRI within a week. Patients were instructed for proper bowel emptying and to be preferably on a liquid-only diet on the day of their scan. They were asked to void urine two hours before the exam. Patients were briefed about the exam and instructed about the various phases of the scan. Training the patient on squeezing, straining, and evacuation is crucial, as their cooperation is paramount for a successful study. Patients were instructed to strain as they would in the lavatory for strain phases. In the evacuation phase, the patients were instructed to strain again till the rectum emptied.

The MRI was done using a 3T MRI scanner (DISCOVERY MR750W, GE Healthcare, Milwaukee, WI) using a 16-channel external phased array coil using various static and dynamic images. The imaging sequences included T2-weighted (T2W) spin echo (SE) sagittal, coronal, and axial images. The slice thickness, field-of-view (FOV), and matrix size for T2W images were 4 mm, 20 cm, and 256 x 224, respectively. Dynamic fast imaging employing steady-state acquisition (FIESTA) images were taken in the rest, strain, and defecation phases. The slice thickness, FOV, and matrix size of dynamic FIESTA images were 10 mm, 36 cm, and 224 x 288, respectively.

The imaging was acquired in the supine position. Approximately 200 ml of warm USG gel was instilled into the rectum to visualize the anorectal junction and rectoceles better, and ~20 ml of USG jelly was instilled into the vaginal canal. Patients were instructed to wear a diaper to prevent spillage.

An additional post-evacuation sequence was added if the patients could not completely evacuate their rectum during the defecation phase. The patients were asked to evacuate in the toilet and, dynamic imaging in maximum straining was taken.

Image analysis

After obtaining an MRI, images were checked for optimal quality. Adequate staining is evident by the whirl of urine in the bladder and movement of the anterior abdominal wall. Static images were evaluated for anatomic defects, ligamentous injuries, and fascial defects. On axial images, the loss of the normal H shape of the vagina indicated paravaginal defects, the integrity of urethral ligaments was documented, and the space of Retzius was assessed for widening. The normal T2 hypointensity of the muscles (puborectalis, iliococcygeus, and external sphincters) was assessed, and any thinning or defect was recorded.

**Figure 2:**
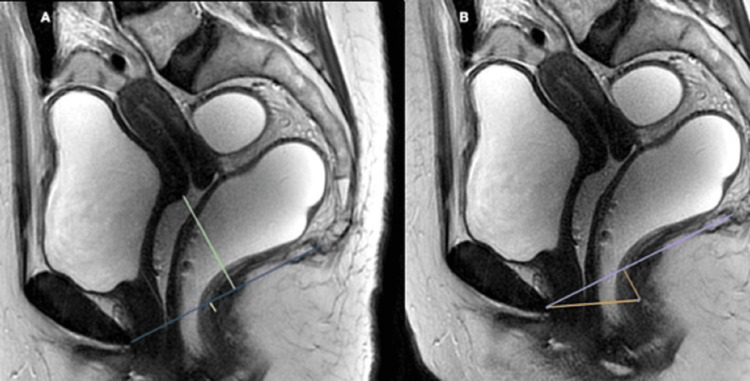
Sagittal T2WI (A) depicting the pubococcygeal line drawn from the inferior aspect of the pubic symphysis to the last coccygeal joint. The most posterior and inferior aspects of the bladder base, the anterior cervical lip, and the posterior wall of the anorectal junction are taken as the reference points in the anterior, middle, and posterior compartments, respectively. The sagittal T2WI (B) depicting the H line (yellow line) and the M line (pink line). T2WI: T2-weighted image.

**Figure 3:**
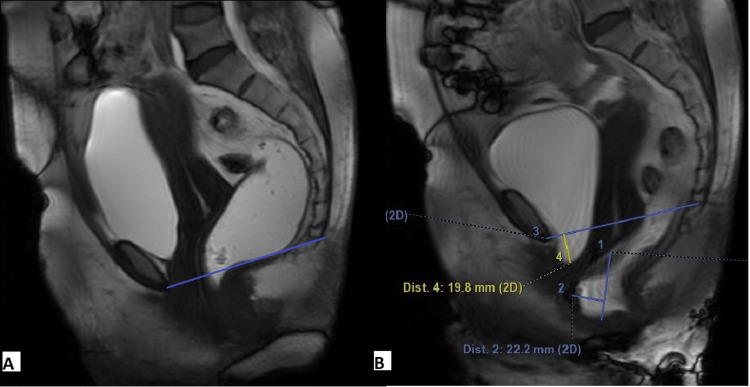
A 48-year-old female complained of stress incontinence and incomplete emptying of the rectum. Sagittal dynamic FIESTA MRI image in rest phase (A) reveals the bladder and anterior cervical lip are above the PCL. Sagittal dynamic FIESTA MRI image in strain phase (B) shows mild cystocele, mild uterine prolapse, and anterior rectocele. FIESTA: fast imaging employing steady-state acquisition; PCL: pubococcygeal line.

Dynamic FIESTA images in the mid-sagittal plane were evaluated to quantify organ prolapse in rest, strain, and defecation phases. The pubococcygeal line (PCL) (drawn from the inferior aspect of the pubic symphysis to the last coccygeal joint) was taken as the reference line. To grade the pelvic floor weakness, H and M lines were used. Measurements were taken in maximum straining phases. The H line was drawn from the inferior aspect of the pubic bone to the posterior wall of the rectum at the anorectal junction. The M line was a perpendicular line drawn from the posterior aspect of the H line to the PCL. The H line represented the anteroposterior width of the levator hiatus, and the M line represented the descent of the pelvic floor. Perpendicular lines were drawn from each compartment's reference points to the PCL in the rest and strain phase. The inferior-most point of the bladder is taken as the reference point in the anterior compartment. The anterior cervical lip is the reference point in the middle compartment and the anterior-most point of the anterior rectocele is in the posterior compartment (Figures 2, 3).

Grading of pelvic organ prolapse was done, as shown in Table 1.

**Table 1:** HMO classification for grading of pelvic floor prolapse. HMO: H-line, M-line, and organ prolapse.

	Small	Moderate	Large
H line	6-8 cm	8-10 cm	>10 cm
M line	2-4 cm	4-6 cm	>6 cm
Cystocele, uterovaginal descent	1-3 cm	3-6 cm	>6 cm
Rectocele	<2 cm	2-4 cm	>4 cm

The overall stage was the highest stage in the anterior, middle, and posterior compartments. For correlation purposes, Aa and Ba were compared to cystocele in MRI, C and D were compared to uterine descent in MRI, Ap and Bp were compared to rectocele on MRI, and overall stage on POP-Q was correlated with the same on MRI. For pelvic floor widening, the H line was correlated with the sum of GH and PB.

Statistical analysis

The interval variables like age, levator plate angle, H line, and M line were summarized by mean as a measure of central tendency and standard deviation as a measure of dispersion. Common measurable parameters between clinical exams and MRI were looked for agreement using kappa statistics and correlation using Spearman correlation coefficient for ordinal variables and Pearson’s correlation coefficient for continuous variables. The correlation coefficient was graded as follows: <0.3 was taken as a weak correlation, 0.3-0.49 = modest, 0.5-0.69 = moderate, and 0.7-0.89 = strong correlation. Cohen’s kappa was graded as 0.2-0.4 = minimal agreement, 0.4-0.6 = weak agreement, 0.6-0.8 = moderate agreement, and >0.8 = strong agreement. P < 0.05 was taken as significant.

## Results

A total of 50 participants were included with a mean age of 50.1 ± 11.4 (range: 23 to 80) years. Most of the patients belonged to the 41-50 years age group. The parity ranged from P1 to P10, with most women having more than four children.

The symptoms ranged from patients complaining of something coming out of the vagina, which was the commonest symptom (96%), followed by stress incontinence (52%), constipation (28%), urge incontinence (22%), and dysuria (16%). Some patients also presented with non-specific symptoms like lower back pain (16%).

The majority of our patients had multicompartment involvement (35, 70%). Anterior compartment involvement was the most common. Cystoceles were found in 45 (90%), urethral hypermobility in 38 (76%), uterine descent in 38 (76%), anorectal junction (ARJ) descent in 43 (86%), anterior rectocele in 42 (84%), and rectal intussusception in 23 (46%) patients. Table 2 shows a descriptive analysis of the various parameters found in dynamic MRI of the pelvic floor.

**Table 2:** Descriptive analysis of the various parameters as found in dynamic imaging of the pelvic floor. LP angle: levator plate angle. The levator plate angle is the angle measured between the levator plate and the pubococcygeal line. ARJ: anorectal junction.

	H line (cm)	M line (cm)	LP angle (degrees)	Bladder descent (cm)	Uterine descent (cm)	Rectocele (cm)	ARJ descent (cm)
Mean	7.310	4.752	42.126	3.9850	2.724	1.912	4.7
SD	1.1488	1.5304	12.3657	2.43956	2.5362	1.233	1.53
Median	7.300	4.800	42.350	3.2000	1.600	1.750	4.8
IQR	6.67-8.20	3.7-5.72	35.0-52.12	2.4-5.42	1.0-3.77	1.17-2.7	3.7-5.7
Minimum	4.6	1.1	8.0	.70	.0	.0	1.1
Maximum	10.5	7.9	69.7	10.60	8.4	4.8	7.9

Clinical and imaging staging and grading of pelvic organ descent were compared. For comparison purposes, various compartment-wise staging was done on POP-Q along with an overall stage. Correspondingly similar staging was done in MRI, considering the points in POP-Q. Table 3 shows the number of patients with different stages and grades in various compartments as detected on POP-Q and dynamic MRI, respectively.

**Table 3:** The number of patients with different stages and grades in various compartments as detected on POP-Q and dynamic MRI, respectively. POP-Q: pelvic organ prolapse quantification.

	POP-Q stages				MRI grades			
	Stage 0	Stage 1	Stage 2	Stage 3	Normal	Mild	Moderate	Severe
Anterior compartment	5	15	14	16	5	18	15	12
Middle compartment	12	23	8	12	12	22	7	9
Posterior compartment	8	5	28	9	8	21	20	1
Overall	1	8	25	16	4	9	22	15

Table 4 shows the correlation between the MRI grading and clinical staging for all three compartments.

**Table 4:** Spearman correlation coefficient between MRI grade and POP-Q stage. POP-Q: pelvic organ prolapse quantification.

		MRI grading			
Clinical stage		Cystocele (grade)	Uterine prolapse (grade)	Rectocele grading	Overall MRI stage
Anterior compartment stage	r value	.871	.762	.062	.500
	p-value	.000	.000	.669	.000
	n	50	50	50	50
Middle compartment stage	r value	.664	.799	-.273	.186
	p-value	.000	.000	.055	.196
	n	50	50	50	50
Posterior compartment stage	r value	.546	.597	.172	.355
	p-value	.000	.000	.233	.012
	n	50	50	50	50
Overall stage	r value	.820	.782	.047	.463
	p-value	.000	.000	.744	.001
	n	50	50	50	50

In the anterior and middle compartments, the correlation coefficient r came out to be 0.871 (p < 0.001) and 0.799 (p < 0.001), indicating a statistically significant strong correlation between the MRI grade and clinical stage. In the posterior compartment, the correlation coefficient (r) was 0.172 (p = 0.233), indicating a statistically insignificant weak correlation. For the overall stage, the correlation was statistically significant and moderate.

## Discussion

Pelvic floor dysfunction is a common problem involving women of all ages, particularly women over 40 years and post-menopausal women [1]. More than 80% of our patients were above 40 years. Multiparity is reported to be one of the strongest predisposing risk factors for primary POP [2,5]. Of our patients, 80% had three or more childbirths.

Similar to other studies [13], the majority of our patients had multicompartment involvement. Identifying diseases of all the compartments is vital before surgery is planned. Studies have shown that in around 30% of cases, anterior or middle compartment descent was associated with rectal intussusception [14]. Failure to identify these pathologies may lead to recurrence.

The findings in our study demonstrated a strong correlation between the MRI grade and clinical stage in the anterior and middle compartments. The correlation between the clinical stage and MRI determined overall grade was moderate. However, in the posterior compartment, there is a weak correlation. Similar results were found in other studies in which a good correlation in the anterior compartment and a poor correlation in the posterior compartment was found [13,19].

In a study conducted by Swamy et al., the correlative measurements of pelvic floor descent by dynamic MRI pelvis study were compared with gynecological POP-Q. In their study, there was a weak correlation between the two in the posterior compartment, which they attributed to the fact the Ap and Bp were arbitrary points and did not correspond to any specific anatomical structure [12]. Difference in the position of examination in MRI and POP-Q is another factor.

**Figure 4:**
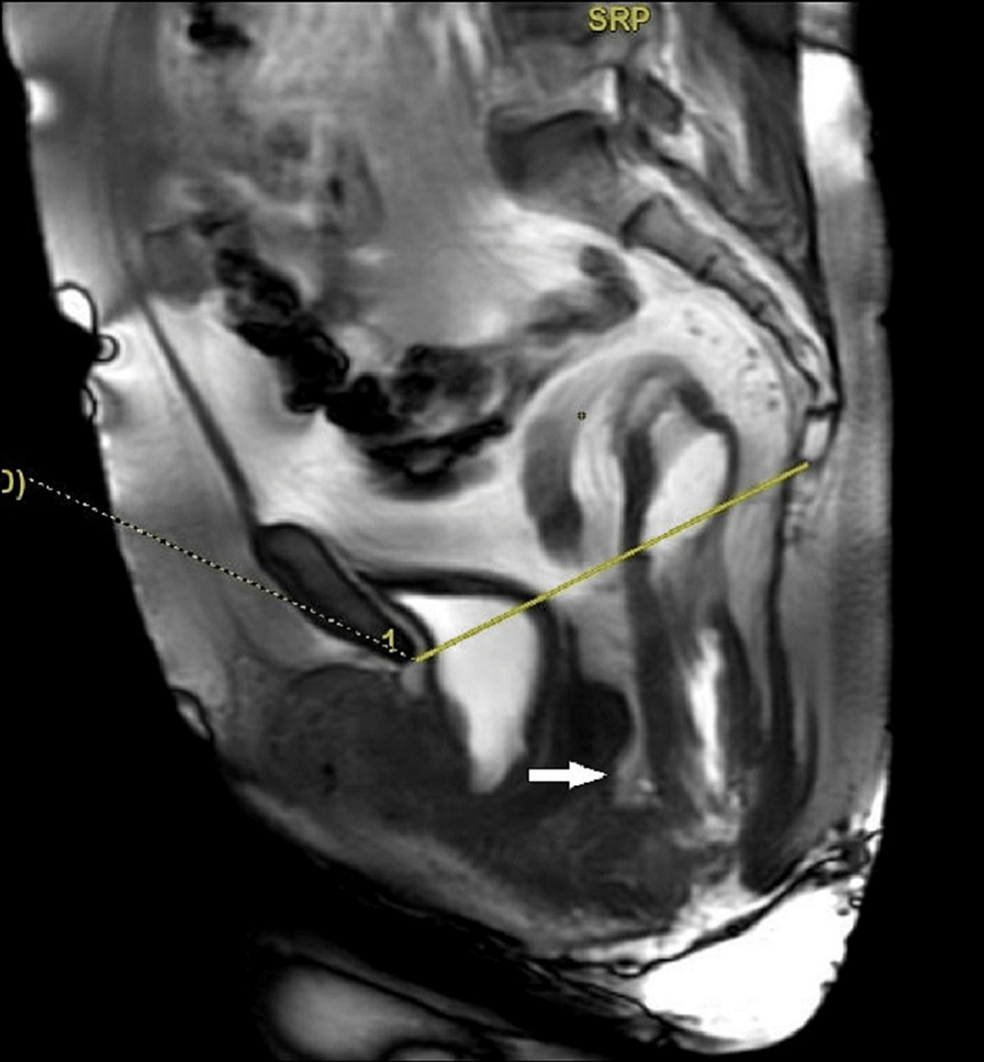
A 53-year-female, post-hysterectomy, with pain during defecation. Midline sagittal dynamic FIESTA MRI image at the end of the defecation phase reveals peritoneocele (white arrow). Also, note the intra-anal rectal mucosa intussusception. FIESTA: fast imaging employing steady-state acquisition.

There was a statistically significant moderate correlation between MRI and clinically assessed levator hiatus. Cul-de-sac hernias are most difficult to identify on clinical examination. Defecation and post-defecation phases in MRI help in the better evaluation of the cul-de-sac hernias (Figure 4). In our study, 17 patients had cul-de-sac hernias (two enteroceles and 15 peritoneoceles), which were best detected on the MRI study at the end of the defecation phases (evacuation phase). As the rectum empties, there is space for the bowel or peritoneum to descend into the cul-de-sac. A study by Poncelet et al. showed 19 peritoneocele and 13 enterocele out of 50 patients on MRI. According to them, MRI in the defecation and evacuation phase performed well in diagnosing cul-de-sac hernias [20]. The differentiation of enterocele and high rectocele can be made on MRI, as it changes the surgical planning. In cases of enterocele, the aim will be to obliterate the cul-de-sac. However, for rectocele, only posterior colporrhaphy suffices [21].

**Figure 5:**
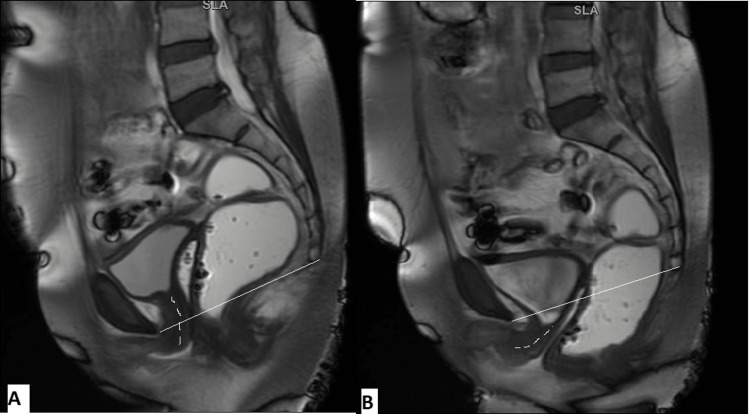
A 45-year-old female complaining of urinary symptoms. (A) Midline sagittal dynamic FIESTA MRI at rest reveals the normal position of the urethra. (B) In sagittal dynamic FIESTA MRI in the maximal strain phase, the urethra is seen inferior to the pubic symphysis in horizontal orientation. Dotted line: urethra; solid line: pubococcygeal line; FIESTA: fast imaging employing steady-state acquisition.

Unmasking of stress urinary incontinence can occur after surgical correction of POP without concomitant incontinence surgery. This is known as de novo stress urinary incontinence and is estimated to be as high as 50% in different studies. It happens due to urethral hypermobility and kinking due to high-grade POP. Preoperative detection of urethral hypermobility and kinking may help adequately manage these patients [22]. Urethral hypermobility was diagnosed in MRI when the urethra was seen in a horizontal orientation and lied inferior to the pubic rami on straining phases (Figure 5).

**Figure 6:**
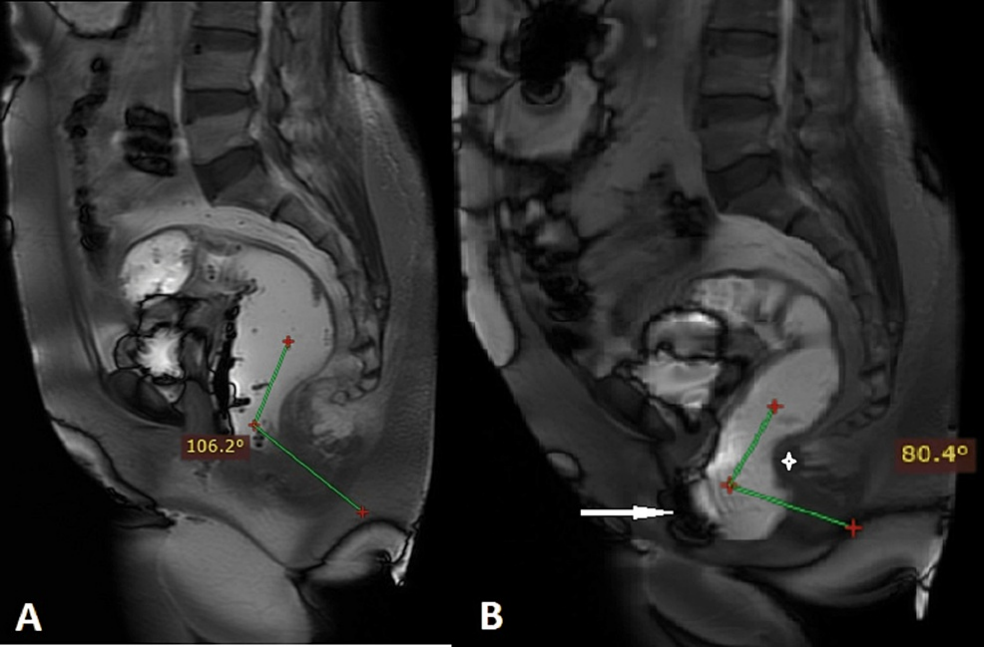
A 58-year-old lady presented with difficulty in defecation and suspected rectocele in clinical examination. Midline sagittal dynamic FIESTA MRI in rest (A) reveals a normal anorectal angle (106°). Midline sagittal dynamic FIESTA MRI in maximal strain (B) reveals a reduction in anorectal angle (80°) instead of an increase with prominent puborectalis sling impression on the posterior rectal wall (asterisk), indicating puborectalis dyssynergia. Anterior rectocele is also noted (white arrow). FIESTA: fast imaging employing steady-state acquisition.

One of our patients with rectocele had obstructed defecation with puborectalis dyssynergia (Figure 6). Identifying pelvic floor dyssynergia in patients with posterior compartment symptoms is important, as they require biofeedback and pelvic floor retraining. Failure to identify this condition when associated with other anatomical abnormalities may result in improper surgery and poor prognosis [23].

**Figure 7:**
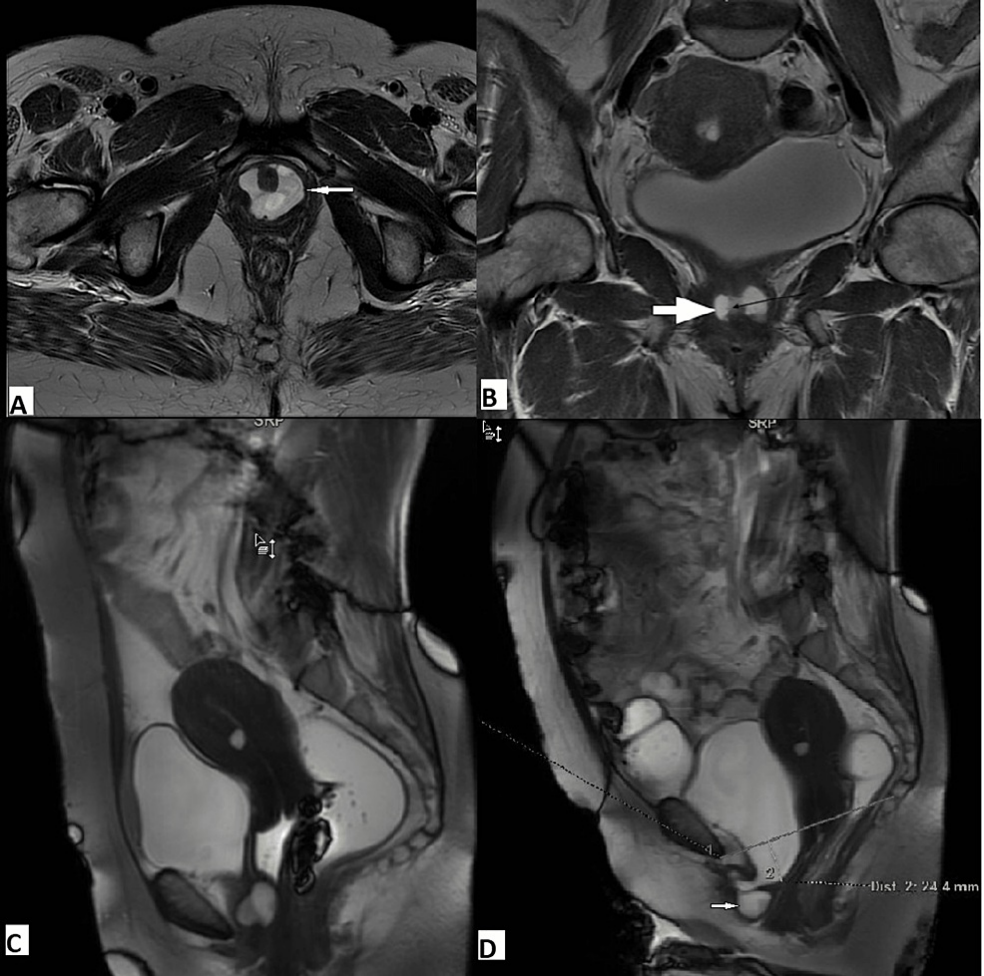
A 46-year-old lady with urethral diverticulum and cystocele. T2 axial (A) and coronal (B) images reveal saddle-shaped fluid collection (white arrow) around the urethra (black arrow), indicating urethral diverticulum. Midline sagittal dynamic FIESTA MRI in rest phase (C) reveals urethral diverticulum with the normal position of the bladder base and urethra. Image in strain phase (D) reveals cystocele with urethral hypermobility. The urethral diverticulum is seen anteriorly (white arrow). FIESTA: fast imaging employing steady-state acquisition.

MRI also reveals associated pathologies that can mimic POP symptoms or change the management approach. Four of our patients had uterine fibroid, and one had a cervical polyp associated with POP. One of our patients with stress incontinence and burning micturition had a mild cystocele and tender lump in the anterior aspect perineum. MRI revealed a grade 1 cystocele with a urethral diverticulum (Figure 7). Similar results were reported in other studies [24,25].

There are a few limitations of our study. Our sample size was relatively small. Further research with larger sample sizes would be beneficial to strengthen the evidence supporting the use of dynamic MRI for evaluating POP.

It cannot be stressed enough that an MRI is done in a supine position, which results in an underestimation of the degree of prolapse. However, studies have found no clinically significant difference between dynamic MRI performed in sitting and supine positions [23,26].

## Conclusions

Our study demonstrated a strong correlation between the MRI grade and clinical stage in the anterior and middle compartment and a weak correlation in the posterior compartment. The correlation between the clinical stage and MRI determined overall grade was found to be moderate. MRI is of utmost importance in posterior compartment prolapse. It adds to the information for better preoperative evaluation of the patient and allows the detection of pathologies, which demands change in the management and surgery. MRI of the pelvic floor can be incorporated into the evaluation of patients with pelvic organ weakness and prolapse and making management decisions, especially in the posterior compartment.
